# Energy Transfer between AGuIX Nanoparticles and Photofrin under Light or X-ray Excitation for PDT Applications

**DOI:** 10.3390/ph17081033

**Published:** 2024-08-05

**Authors:** Batoul Dhaini, Philippe Arnoux, Joël Daouk, François Lux, Olivier Tillement, Agnès Hagège, Tayssir Hamieh, Gal Shafirstein, Céline Frochot

**Affiliations:** 1 Université de Lorraine, CNRS, LRGP, 54600 Nancy, France; batoul.dhaini@outlook.com (B.D.); philippe.arnoux@univ-lorraine.fr (P.A.); 2 Laboratory of Materials, Catalysis, Environment and Analytical Methods (MCEMA), Faculty of Sciences I, Lebanese University, Beirut 1102, Lebanon; 3 Université de Lorraine, CNRS, CRAN, 54505 Vandoeuvre-les-Nancy, France; joel.daouk@univ-lorraine.fr; 4 Université de Lyon, CNRS, ILM, 69007 Lyon, France; francois.lux@univ-lyon1.fr (F.L.); olivier.tillement@univ-lyon1.fr (O.T.); 5 Université Claude Bernard Lyon 1, CNRS, ISA, 69100 Villeurbanne, Francetayssir.hamieh@ul.edu.lb (T.H.); 6 Faculty of Science and Engineering, Maastricht University, 6211 LK Maastricht, The Netherlands; 7 Roswell Park Comprehensive Cancer Center, Buffalo, NY 14226, USA; gal.shafirstein@roswellpark.org

**Keywords:** PDT, Photofrin, FRET, cancer, AGuIX nanoparticles

## Abstract

Photodynamic therapy is an accepted therapy cancer treatment. Its advantages encourage researchers to delve deeper. The use of nanoparticles in PDT has several advantages including the passive targeting of cancer cells. The aim of this article is to evaluate the effectiveness of AGuIX nanoparticles (activation and guiding of irradiation by X-ray) in the presence or absence of a photosensitizer, Photofrin, under illumination of 630 nm or under X-ray irradiation. The goal is to improve local tumor control by combining PDT with low-dose-X-ray-activated NPs in the treatment of locally advanced metastatic lung cancer. The study of the energy transfer, which occurs after excitation of Gd/Tb chelated in AGuIX in the presence of Photofrin, was carried out. We could observe the formation of singlet oxygen after the light or X-ray excitation of Gd and Tb that was not observed for AGuIX or Photofrin alone, proving that it is possible to realize energy transfer between both compounds.

## 1. Introduction

Photodynamic therapy (PDT) is an anti-bacterial and anti-cancer treatment technique based on the combination of a photosensitive molecule called a photosensitizer (PS) capable of internalizing into cells and generating ROS including ^1^O_2_ upon activation of light and interaction with oxygen. The ^1^O_2_ and other ROS produced are cytotoxic species that react with cellular components and induce biochemical disorders in the cell. These localized photophysical processes and photochemical reactions are at the core of PDT. Thus, light, PS, and oxygen are the essential components of PDT. Separately, these components do not exhibit any toxicity, and the absence of any of them eliminates the photodynamic effect. It is the combination of these three elements, with well-tuned light and PS dosimetry, that determines the overall effectiveness of PDT [1,2]. Usually, the PDT causes cellular death by necrosis [3].

**Figure 1:**
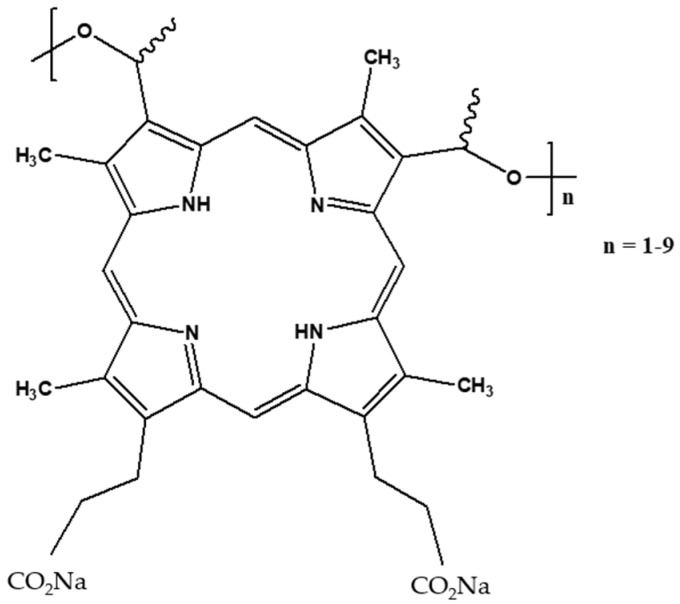
Chemical structure of Photofrin^®^.

Photofrin is a mixture of several porphyrins. It is a porfimer sodium used clinically in PDT for the treatment of different types of cancers such as esophageal cancer and endobronchial cancer. The structure of Photofrin is given in Figure 1. Photofrin is approved in the United States to treat esophageal and lung cancer [4]. The quantum yield of ^1^O_2_ production after the excitation of Photofrin is 0.8 in ethanol and 0.15 in D_2_O [5]. In [6], Wilkinson et al. also reported the quantum yield of ^1^O_2_ production for many photosensitizers. The ^1^O_2_ production quantum yield of Photofrin was found to be between 0.01 and 0.13 depending on the technique used to perform the calculation. In water, ^1^O_2_ has a lifetime of around 3.5 μs. In biological medium, ^1^O_2_ presents a shorter lifetime and can react only with the biomolecules close to it. It is reported that the intracellular lifetime is around 3 µs [7,8] but was initially reported to be 0.04%µs [9,10]. This allows us to estimate the diffusion distance using the equation d = (6Dt)^0.5^, where d is the diffusion distance that ^1^O_2_ can move in a period of time t, and D is the diffusion coefficient (2–4 × 10^−6^ cm^2^ s^−1^). The diffusion distance is around 100 nm (against the 20 nm previously estimated).

In clinical applications, the excitation of Photofrin is performed with red light in order to obtain the best penetration of light, knowing that the absorption of light by the skin and blood limits its penetration to a depth not greater than 2 mm [11]. To overcome the light penetration problem, it is possible to use X-rays in PDT (PDT-X) [12]. The principle of PDT-X is to use X-ray instead of light. X-ray is used to excite a radiosensitizer or a specific nanoparticle, which will emit light that will then be absorbed by the photosensitizer to generate the PDT effect. This concept was developed first by Chen et al. in 2006 [13]; now, more and more publications and journals are taking an interest in PDTX [12,14,15,16,17,18,19,20].

AGuIX NPs (Activation and Guidance of Irradiation by X-ray Nanoparticles) were developed by NH TherAguix to improve the efficacy of radiotherapy in the treatment of cancer. These nanoparticles are made of a polysiloxane core coupled with a chelating agent with gadolinium. AGuIX^®^ concentrates a high number of gadolinium atoms (∼15) in an ultrasmall object (∼5 nm). They are being evaluated in five clinical trials: NANORAD 2 for the treatment of brain metastases; NANOCOL, a Phase 1b trial in combination with cisplatin-based chemo-radiotherapy followed by brachytherapy for the treatment of locally advanced cervical cancer; NANOBRAINMETS, a Phase 2 trial evaluating AGuIX^®^ for the treatment of brain metastases by stereotactic irradiation; NANOSMART, a Phase 1b/2 trial to evaluate AGuIX in combination with stereotactic magnetic resonance-guided adaptive radiation therapy (SMART) for the treatment of locally advanced or unresectable pancreatic cancers and centrally located lung cancers/metastasis; NANOGBM, a Phase 1b/2 multicentric trial for the treatment of newly diagnosed glioblastome.

In this study, we investigated the energy transfer between the gadolinium chelated into AGuIX nanoparticle and Photofrin. We also replaced gadolinium with terbium and evaluated the energy transfer between the terbium chelated into AGuIX nanoparticle and Photofrin. We studied the difference of the energy transfer when Photofrin was either in solution or bound without any chemical reaction to the surface of AGuIX by adsorption. After AGuIX irradiation, we showed there was production of ^1^O_2_ due to a Förster Resonance Energy Transfer (FRET) between AGuIX and Photofrin.

## 2. Results and Discussion

AGuIX-Tb and AGuIX-Gd have been obtained by a synthesis already described in the literature [21]. Tb_2_O_3_ or Gd_2_O_3_ cores are obtained by the addition of soda on terbium chloride or gadolinium chloride dissolved in diethylene glycol. Then, growth of a polysiloxane shell is realized by the addition of two silane precursors in diethylene glycol (i.e., (3-aminopropyl)triethoxysilane and tetraethoxysilane). DOTAGA is then grafted on the inorganic matrix by the reaction of DOTAGA anhydride on the free amino functions. Acetone is then added to precipitate the nanoparticles before their dispersion in water. During the passage to water, the lanthanide oxide core is dissolved, and the lanthanide ions (i.e., terbium or gadolinium) are chelated by DOTAGA leading to the fragmentation of the polysiloxane shell in the final ultrasmall AGuIX nanoparticles [22,23]. The whole mixture is then purified by tangential filtration to remove the polysiloxane fragments and lanthanide ions before freeze drying.

### 2.1. Photophysical Properties of Photofrin in Water

**Figure 2:**
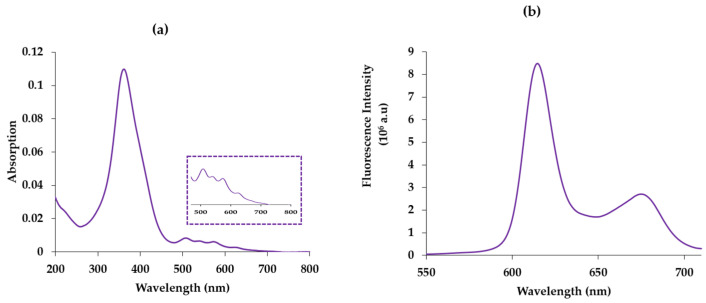
(**a**) Absorption spectrum of Photofrin in water. [Photofrin] = 2.5 µM. ε505 nm (Q_IV_) = 9544 cm^−1^ M^−1^, ε540 nm (Q_III_) = 8084 cm^−1^ M^−1^, ε559 nm (Q_II_) = 7635 cm^−1^ M^−1^, ε612 nm (Q_I_) = 3593 cm^−1^ M^−1^, (**b**) emission spectrum of Photofrin in water. [Photofrin] = 25 μM, λ_excitation_ = 366 nm.

To evaluate the energy transfer between the donor (AGuIX) and the acceptor (Photofrin), we first studied the photophysical properties of Photofrin alone in water. The photophysical characteristics of Photofrin are similar to those of porphyrins. Its absorption maximum (Soret band) is at 366 nm, and like all porphyrins, it owns four bands (Q_I–IV_), which are located at 507 nm, 540 nm, 568 nm and 618 nm, respectively, with a maximum extinction coefficient (λ = 366 nm) of 147,206 M^−1^ cm^−1^ (Figure 2a). After excitation of Photoforin at 366 nm in the Soret band, the fluorescence emission spectrum presents two emission bands at 614 nm and 676 nm (Figure 2b). It is in agreement with the spectrum obtained in [5]. These two bands are due to the radiative deactivation from the Photofrin singlet state S_1_ to the Photofrin fundamental state S_0_.

### 2.2. Study of the Energy Transfer between Free Lanthanides and Chelated Lanthanides in AGuIX in Solution

#### 2.2.1. TbCl_3_/Photofrin and AGuIX Tb/Photofrin

**Figure 3:**
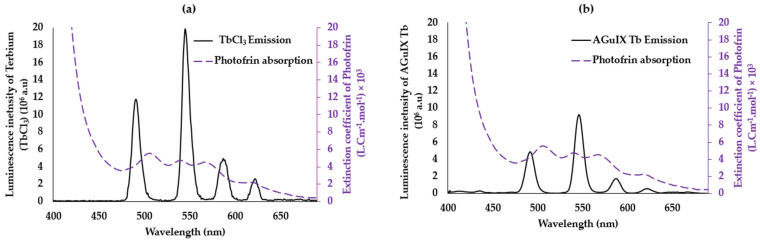
(**a**) Overlap between the emission spectrum of TbCl_3_ and the absorption spectrum of Photofrin. (**b**) Overlap between the emission spectra of AGuIX Tb and absorption of Photofrin (violet) in water. λ_excitation_ = 351 nm, delay 50 μs.

In order to observe the transfer of energy between the donor (lanthanides) and the acceptor (Photofrin), it is already necessary to have energy compatibility between the donor and the acceptor, which is represented by the overlap between the emission spectrum of the donor and the absorption of the acceptor. We recorded the emission spectrum of TbCl_3_ used as a model molecule and the absorption spectrum of Photofrin (Figure 3a). The four terbium emission peaks at 488 nm, 545 nm, 585 nm, and 620 nm correspond to the electronic transitions between the ^5^D_4_ level and ^7^F_6_, ^7^F_5_, ^7^F_4_, and ^7^F_3_, respectively. We evaluated the overlap J to be 8.9 × 10^14^ (M^−1^ nm^4^ cm^−1^) (Equation (2)). This overlap allows us to calculate the Forster radius for which the energy transfer efficiency is 50% (Equation (3)), R_0_ = 3.33 nm.

We recorded the emission spectrum of the terbium chelated in the DOTA of the AGuIX nanoparticles and the absorption spectrum of Photofrin (Figure 3b). The same four terbium emission bands could be observed; the chelation does not change the maximum of emission even if the intensity seems to be lower. The overlap J is equal to 1.12 × 10^15^ (M^−1^ nm^4^ cm^−1^). This overlap is higher than in the case of TbCl_3_ (Figure 2b); the R_0_ was found to be 3.46 nm, with a slight difference compared to the unchelated Tb in AGuIX (R_0_ = 3.33 nm). These results show the possibility of having a non-radiative FRET energy transfer between the two pairs with an efficiency of the AGuIX Tb@Photofrin pair greater than that of TbCl_3_@Photofrin because R_o(AGuIX Tb@Photofrin)_ > R_0(TbCl3@Photofrin)_.

**Figure 4:**
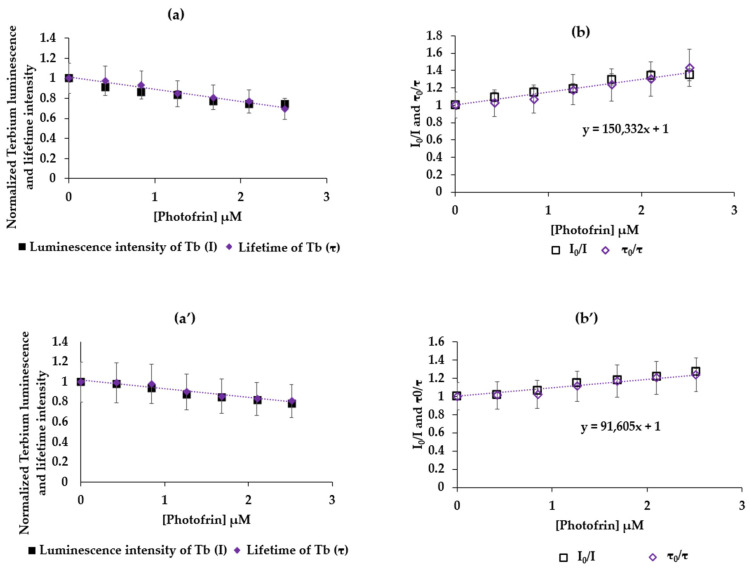
(**a**) TbCl_3_ I = f(Photofrin]) and τ = f(Photofrin]), (**b**) I_0_/I = f([Photofrin]) and τ_0_/τ = f([Photofrin]) in water, correlation = 0.943. λ_excitation_ = 351 nm, 50 μs delay. AGuIX Tb (**a’**) I = f(Photofrin]) and τ = f(Photofrin]), (**b’**) I_0_/I = f([Photofrin]) and τ_0_/τ = f([Photofrin]) in water correlation = 0.987, λ_excitation_ = 351 nm, 50 μs delay.

To evaluate whether there is a transfer or not, we studied the variation in the luminescence intensity (I) and the luminescence lifetime (τ) of terbium, as a function of the concentration of Photofrin with a fixed concentration of Tb ([Tb] = 10 mM). Figure 4 shows (a) I = f([Photofrin]) and τ = f([Photofrin]) and (b) I_0_/I = f([Photofrin]) and τ_0_/τ = f([Photofrin]) in water with λ_excitation_ = 351 nm and a delay of 50 μs. Assuming that n = 1, the molar mass of Photofrin is considered equal to 1179 g/mol.

Figure 4 shows for TbCl_3_ (a and b) and AGuIX Tb (a’ and b’) an increase in I_0_/I = f([Photofrin]) and τ_0_/τ = f([Photofrin]) confirming the FRET energy transfer with dynamic inhibition. Knowing that y = F/F_0_ and x = [Photofrin], the slope is the Stern–Volmer constant. Since the luminescence lifetimes of Tb (TbCl_3_) and AGuIX Tb are 433 µs and 2.4 ms, respectively, Kq is found to be equal to and 3.6 × 10^8^ M^−1^ s^−1^ and 4.3 × 10^7^ M^−1^ s^−1^, respectively, and K_SV_ is found to be equal to 1.5 × 10^5^ M^−1^ and 1.05 × 10^5^ M^−1^ (Equation (4)), respectively. The excitation of TbCl_3_ or AGuIX Tb in the presence of Photofrin at 351 nm induces a FRET.

#### 2.2.2. GdCl_3_/Photofrin and AGuIX Gd/Photofrin

**Figure 5:**
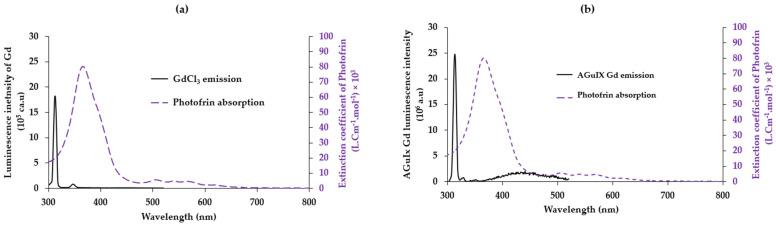
(**a**) Overlap between the emission of GdCl_3_ in water excited at 273 nm and the absorption spectrum of Photofrin in water. (**b**) Overlap between the emission spectrum of AGuIX Gd and the absorption spectrum of Photofrin in water. Gd is excited at 273 nm.

The same studies were carried out for the couple, gadolinium and Photofrin, as well as gadolinium chelated in AGuIX nanoparticles and Photofrin (Figure 5). The emission of Gd at 313 nm corresponds to the energy difference between the ^6^P_J_ and ^6^S_7/2_ energy levels. Gadolinium is the only one of the lanthanide group, which has a first energy state that is too high, which justifies the unique narrow emission peak. A similar emission peak was observed for GdCl_3_ and Gd chelated in AGuIX. The overlap between the emission spectrum of free gadolinium and the absorption spectrum of Photofrin is J = 3.433854 × 10^14^ (M^−1^ nm^4^ cm^−1^) with R_0_ = 2.8 nm (Figure 5a); whereas, the overlap is J = 5.425214 × 10^14^ (M^−1^ nm^4^ cm^−1^), and the Forster radius R_0_ = 3.06 nm (Figure 4b) for the couple AGuIX Gd and Photofrin. This could indicate a higher FRET efficiency between AGuIX Gd and Photofrin than between the GdCl_3_ and Photofrin.

**Figure 6:**
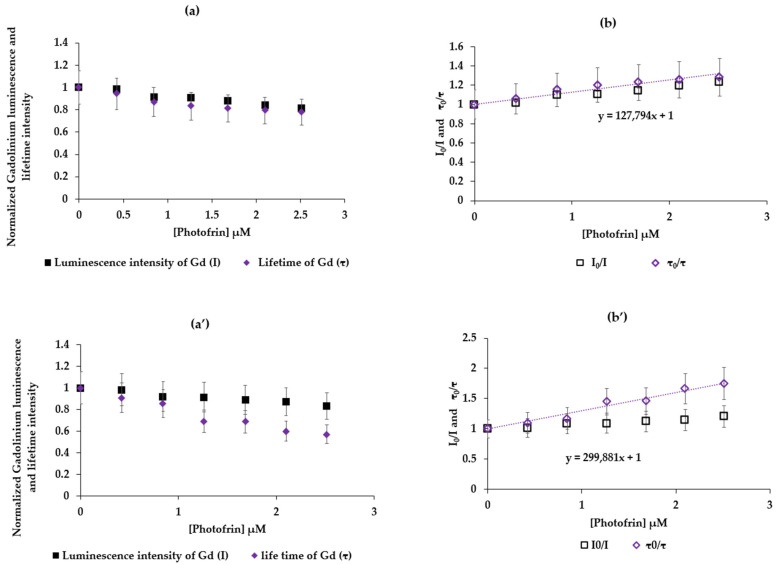
(**a**) I = f([Photofrin]) and τ = f([Photofrin]). (**b**) I_0_/I = f([Photofrin]) and τ_0_/τ = f([Photofrin]) in water, correlation = 0.961. λ_excitation_ = 273 nm—delay 50 μs. (**a’**) I = f([Photofrin]) and τ = f([Photofrin]), (**b’**) I_0_/I = f([Photofrin]) and τ_0_/τ = f([Photofrin]) in water, correlation = 0.950, λ_excitation_ = 273 nm, delay 50 μs.

We present in Figure 6a I = f([Photofrin]) and τ = f([Photofrin]) and Figure 6b I_0_/I = f([Photofrin]) and τ_0_/τ = f([Photofrin]) in water, λ_excitation_ = 273 nm with a delay of 50 μs. For the couple GdCl_3_ and Photofrin and, likewise, (a’) and (b’) for AGuIX Gd and Photofrin, an increasing linearity of I_0_/I = f([Photofrin]) and τ_0_/τ = f([Photofrin]) confirms a FRET energy transfer with the dynamic inhibition between Gd and Photofrin. Since the luminescence lifetimes of Gd (GdCl_3_) and AGuIX Gd are 500 µs and 2 ms, respectively, K_q_ is found to be equal to 2.4 × 10^8^ M^−1^ s^−1^ and 1.4 × 10^8^ M^−1^ s^−1^, respectively, and K_SV_ is found to be equal to 1.2 × 10^5^ M^−1^ and 2.9 × 10^5^ M^−1^, respectively.

### 2.3. Study of the Energy Transfer between Photofrin Adsorbed on AGuIX Gd Nanoparticles

#### 2.3.1. Energy Transfer between AGuIX Gd and Photofrin Adsorbed on AGuIX Gd

After adsorption of AGuIX with Photofrin (see Materials and Methods), the four batches of the resulting nanoparticles on which Photofrin was adsorbed show an increase in the zeta potential in absolute value compared to AGuIX alone. The zeta potential of AGuIX Gd@0.1 Photofrin, AGuIX Gd@0.075 Photofrin, AGuIX Gd@0.05 Photofrin, and AGuIX Gd@0.025 Photofrin were −17.8 mV, −19.8 mV, −22.1 mV, and −23.5 mV, respectively, compared to 0.377 mV for AGuIX Gd alone, at a pH equal to 7. AGuIX Gd@0.1 Photofrin is significantly larger than the others due to a greater amount of Photofrin, which increases the repellency due to the COO-terminal function. The size of the AGuIX Gd@Photofrin NPs shows two populations: AGuIX Gd@0.1 Photofrin (population 1:1.4 nm (74%), 3.9 nm (26%)), AGuIX Gd@0.075 Photofrin (population 1:1.5 nm (63%), 5 nm (37%)), AGuIX Gd@0.05 Photofrin (population 1:1.4 nm (75%), 4.5 nm (25%)), and AGuIX Gd@0.025 Photofrin (population 1:1.2 nm (63%), 4 nm (37%)), by the TDA-ICP method.

**Figure 7:**
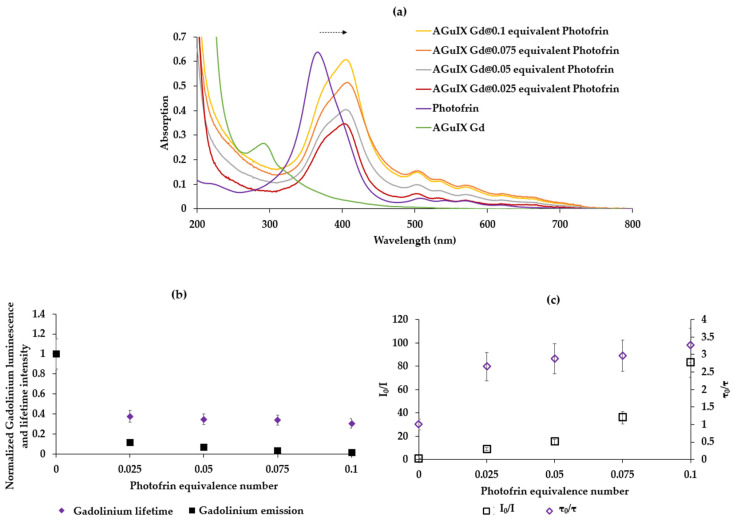
(**a**) Absorption spectra of AGuIX Gd NP, Photofrin, and Photofrin adsorbed on AGuIX in water (**b**) Normalized Gd luminescence and lifetime intensity in function of Photofrin equivalent number adsorbed on AGuIX (**c**) I_0_/I = f([Photofrin]) and τ_0_/τ = f([Photofrin]) in water, correlation = 0.662, λ_excitation_ = 273 nm, delay 50 μs.

The absorption spectra of AGuIX Gd NP, Photofrin, and Photofrin adsorbed on AGuIX are reported in Figure 7a. A 38 nm batochrome shift of the Soret band is observed when Photofrin is adsorbed onto the NPs compared to Photofrin alone in solution, indicating successful adsorption. Figure 7 shows (b) I = f([Photofrin]) and τ = f([Photofrin]) (c) I_0_/I = f([Photofrin]) and τ_0_/τ = f([Photofrin]) in water after excitation at 273 nm with a delay of 50 μs. The concentrations of Photofrin are expressed in the molar mass concentration of equivalent relative to Gd.

The shape of the graphs in Figure 7b,c indicates a FRET energy transfer with dynamic and static inhibition, since I_0_/I is exponential, and τ_0_/τ is linear.

In order to check whether there is ^1^O_2_ production after the energy transfer between AGuIX Gd NP and Photofrin, we recorded the ^1^O_2_ emission spectrum after the excitation of AGuIX Gd NP coated by Photofrin. First of all, AGuIX Gd without Photofrin did not produce ^1^O_2_ after excitation at 273 nm in D_2_O. The ^1^O_2_ quantum yields with 0.1, 0.075, 0.05, and 0.025 molar equivalents of Gd were calculated and were 21%, 25%, 32%, and 32%, respectively. It appears that the higher the concentration of Photofrin, the lower the ^1^O_2_ quantum yield. This might be due to the aggregation of Photofrin if the concentration is too high.

#### 2.3.2. X-ray Excitation in Solution

**Figure 8:**
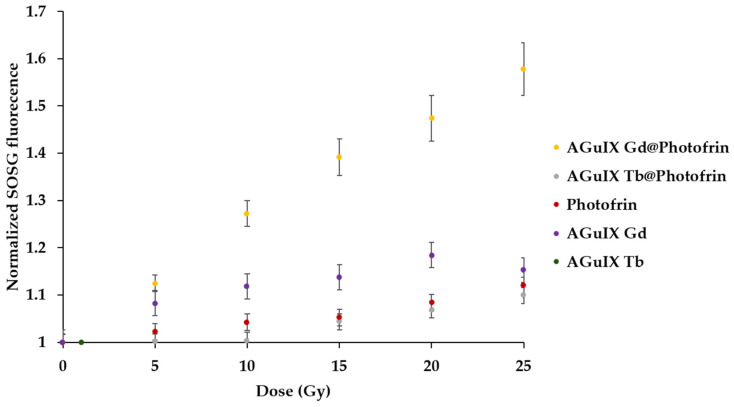
SOSG fluorescence after excitation of AGuIX Gd@Photofrin (0.025 equivalent), AGuIX Tb@Photofrin (0.025 equivalent), Photofrin, AGuIX Tb, and AGuIX Gd (320 kV/10 mA).

Through the experiments performed with AGuIX Gd NP adsorbed with Photofrin, we concluded that the best concentration of Photofrin was 0.025 equivalent of Gd. After excitation by X-rays, we were unable to measure the luminescence of ^1^O_2_ directly because the set-up did not allow us to do so. In this case, we chose to use the SOSG (Singlet Oxygen Sensor Green) probe. An increase in the SOSG fluorescence indicates the formation of ^1^O_2_. The SOSG fluorescence after X-ray irradiation (320 kV/10 mA) of Photofrin, AGuIX Tb, AGuIX Gd, AGuIX Gd@0.025 Photofrin, and AGuIX Tb@0.025-adsorbed Photofrin is presented in Figure 8. The energy transfer was confirmed between Gd and Photofrin under X-rays in solution without a significant production of ^1^O_2_ for the other NPs as well as Photofrin alone.

The Kruskal–Wallis test revealed a significant difference between the slope of the different assessed samples. The post-hoc test indicated the AGuIX Gd@Photofrin sample was significantly different from all the other samples (*p* < 10^−6^). In addition, AGuIX Gd alone was also found different from the control samples (*p* < 10^−4^). This could be attributed to a direct interaction between Gd luminescence and the SOSG probe.

AGuIX nanoparticles accumulate mainly in cancer cells via the enhanced permeability retention (EPR) effect. Hence, this strategy offers two main benefits: first, by the presence of high Z material in the nanoparticle, energy deposition in increased in the nanoparticle neighborhood (radiopotentialization). Thus, the total dose delivered to healthy tissue can be lowered, while keeping the same deposited dose in the tumor cells. Second, due to the PS adsorbed on the nanoparticle, the energy transfer from lanthanide to Photofrin yields a PDT effect by producing singlet oxygen. Hence, the current trend aims at lowering the total X-ray dose by taking advantage of these two effects [24].

In our team, we have already designed nanoparticles for PDTX; we used porphyrin [25,26], chlorin, and phthalocyanine [27]. In this study, we wanted to be as close as possible to clinical use, by using a PS already used in clinics and nanoparticles in clinical phase II. We proved that energy transfer could be possible between lanthanide and Photofrin after the light or X-ray excitation of lanthanide, which was already shown in different studies. For example, E. Abliz et al. synthesized a 20-micron particle Tb(15–20%):Gd_2_O_2_S, which was injected with Photofrin into glioblastoma cells. Knowing that, clinically, 1 to 2 mg of Photofrin/kg of the human body can be injected and assuming a mass of 50 kg, Abliz et al. used 20 µg/mL of Photofrin and 5 mg/mL of Tb/Gd_2_O_2_S, excited via X-ray (120 kVp, 20 mA). More than 90% of the glioblastoma cells were destroyed [28]. Li Wang et al. studied the energy transfer between quantum dots (QD) with a CdSe core and a ZnS shell and Photofrin. After covalently coupling Photofrin on functionalized QD, the energy transfer efficiency was shown to be 100%, after incubating Photofrin/QD (291:1) in H460 cells. The cell death was greater with Photofrin/QD under 6 MV in comparison with cells treated by QD or radiation alone [29,30]. Kulka et al. carried out several studies, which showed that Photofrin alone can also be a selective radiosensitizer with high activity for the treatment of cancer [31]. In 2001, by injecting 10 mg/kg of Photofrin and irradiating over 5 Gy a RT4 bladder cancer cell line implanted in nude mice, the cancer doubling time was reduced by 4 days [31]. They demonstrated that the tumor response was maximal for only 7.5 mg/kg and, irradiating with 3 Gy, the volume of a Lewis sarcoma tumor implanted in Balb/c mice in 6 days decreased by 50% [32]. This strategy of using X-ray instead of light in PDT is growing rapidly, as can be seen from the many recent reviews written on the subject, especially by using nanoparticles [16,17,18].

## 3. Materials and Methods

### 3.1. Materials

Ultrapure water (Milli–Q, ρ > 18 MΩ⋅cm) was used in all the experiments. Dichloromethane (DCM), dimethylformamide (DMF), and ethanol (EtOH) were obtained from Sigma-Aldrich, Saint Louis, MO, USA and used without further purification, as were Terbium (III) chloride hexahydrate (99.9%), Chloride Gadolinium (III) hexahydrate (99.9%), Photofrin (CHRU Lille), and AGuIX (NH TherAguix, Meylan, France).

Absorption spectra were recorded on a UV-3600 UV–visible double beam spectrophotometer (Shimadzu, Marne-La-Vallée, France). Fluorescence spectra were recorded on a Fluorolog FL3-222 spectrofluorometer (Horiba Jobin Yvon, Palaiseau, France) equipped with a 450 W Xenon lamp and thermostatic cell compartment (25 °C), a UV–visible photomultiplier R928 (Hamamatsu Photonics, Hamamatsu, Japan), and an InGaAs infrared detector (DSS-16A020L Electro-Optical System Inc., Phoenixville, PA, USA). The excitation beam was diffracted by a double-ruled grating SPEX monochromator (1200 grooves/mm blazed at 330 nm). The emission beam was diffracted by a double-ruled grating SPEX monochromator (1200 grooves/mm blazed at 500 nm). Singlet oxygen emission was detected through a double-ruled grating SPEX monochromator (600 grooves/mm blazed at 1 µm) and a long-wave pass (780 nm). All spectra were measured in four-face quartz vials. All the emission spectra (fluorescence and singlet oxygen luminescence) have been displayed with the same absorbance (less than 0.2) with the lamp and photomultiplier correction.

The spectral overlap, as well as the Förster radius, were computed to characterize the energy transfer from the Tb or Gd cation (Tb^3+^ or Gd^3+^) to Photofrin. Moreover, the Tb luminescence decay profile was recorded using a Fluorolog spectrofluorometer; the excitation wavelength was set at 351 nm, and the emission peaks were scanned in the 400–690 nm region. The luminescence lifetime of Tb alone or in mixture with P1 was recorded using the lifetime Fluorolog. We assessed the 545 nm peak decay, as it is the highest Tb fluorescence peak. If relevant, we computed the quenching constant (expressed as L mol^−1^ s^−1^) as Kq = K_SV_/τ_0_, where K_SV_ is the Stern–Volmer constant, which was graphically determined; τ_0_ is the Tb fluorescence lifetime without a photosensitizer.

TDA experiments were conducted using a TDA-ICP-MS hyphenation between a Sciex P/ACE MDQ instrument and 7700 Agilent ICP-MS. Fused silica capillaries with an inner diameter of 75 µm and outer diameter of 375 µm and a total length of 64 cm were coated with hydroxypropylcellulose (HPC) using a solution of 0.05 g mL^−1^ in water. Detection was carried out by ICP-MS at m/z = 158 with a data acquisition rate of 500 ms point-1. The samples were hydrodynamically injected (0.3 psi for 3 s) and then mobilized using Tris 10 mM and NaCl 125 mM at 0.7 psi. Between runs, the capillary was flushed at 5 psi for 5 min with the mobilization medium. The detected peak was then fitted by a sum of Gaussian distributions using Origin 8.5 software, according to the following equation:

(1) $$S\left(t\right)=\sum_{i=1}^{2}\left(\frac{A_{i}}{\sigma_{i\;\sqrt{2\pi }}}\right)\exp-\frac{{\left(t-t_{0}\right)}^{2}}{2\sigma_{i}^{2}}$$

where *t*_0_ is the peak residence time, and *σi* and *Ai* are the area under the curve and the temporal variance associated with each species i, respectively. The reference to evaluate the ^1^O_2_ quantum yield after excitation of Photofrin is methylene blue in D_2_O (0.52) [33].

### 3.2. Formatting of the Mathematical Components


(2) $$J=\int f_{D}\left(\lambda \right)\varepsilon_{A}\left(\lambda \right)\lambda^{4}d\lambda$$

(3) $${R_{0}=0.02108\;[\kappa^{2}\Phi_{D}n^{-4}J_{\left(\lambda \right)}]}^{1/6}$$

(4) $$\frac{I_{0}}{I}=1+K_{SV}[Q]$$

(5) $$K_{SV\;}=K_{q\;}\tau_{0}$$

- R_0_, the Forster radius, is the donor–acceptor distance for which the energy transfer efficiency is 50%.
- Quenching constant (k_q_).
- Stern–Volmer constant (K_SV_/slope of curve I_0_/I = f([Quencher]).
- τ_0_ is the luminescence lifetime of the donor without any quencher.

### 3.3. Adsorption of Photofrin on AGuIX Gd

In order to increase the energy transfer between lanthanide and Photofrin, we decided to adsorb the Photofrin beforehand on the AGuIX Gd nanoparticles in order to reduce the distance between Photofrin and Gd. We prepared the solutions by dispersing 10 mM of AGuIX Gd in 800 μL of water for one hour in order to have good stability and dispersion. This solution was divided into four, and we added to each of the solutions, respectively, 0.1 (0.8 μmol), 0.075 (0.6 μmol), 0.05 (0.4 μmol), and 0.025 (0.2 μmol) equivalent of Photofrin in water, drop by drop. These solutions were left stirring for 1 h at ambient temperature. Centrifugation was carried out with vivaspin^®^ 5 kDa MWCO until a transparent filtrate was obtained (followed by absorption until the detection of Photofrin absorption stopped). We checked that the filtrate contained no trace of Photofrin, and the adsorption was complete. The solutions were then lyophilized.

### 3.4. Singlet Oxygen Production during X-ray Irradiation

The synthesis of AGuIX Gd has already been described in the literature [21]. The same protocol was used to elaborate AGuIX Tb by using TbCl_3_ instead of GdCl_3_. The reaction mixture was prepared in 30 mM Tris/HCl (pH 7.4) containing 400 µM AGuIX Tb, AGuIX Gd, AGuIX Gd@0.025 Photofrin, and AGuIX Tb@0.025-adsorbed Photofrin and a 10 µM SOSG probe. Singlet oxygen quenching was achieved by the addition of NaN_3_ (stock solution, 1 M) prepared in the same buffer, to a final concentration of 10 mM. Irradiations were performed on the OptiRAD platform on an XRAD-320 irradiator (Precision X-rays Inc. Madison, CT, USA). The tube settings were set to 320 kVp and 12.5 mA, and the source–surface distance was adjusted to yield a 3.0 Gy/min dose rate. Indeed, as we previously demonstrated a linear relationship between the kV X-ray generator setting and scintillator luminescence intensity, we used the highest voltage available on the XRAD-320 device (i.e., 320 kV), and then, the current and source–surface distance were adjusted to yield the desired dose rate [26].

The fluorescence emission was detected spectroscopically at 525 nm for SOSG. Home-made software allowed long acquisition times and the synchronization between the laser illumination and the signal recording. The integration time was set to 100 ms, and time points were acquired each 5 Gy from 0 to 25 Gy. Moreover, Photofrin at 100 µM was irradiated without nanoscintillator with the same parameters to validate the absence of their direct excitation by X-rays.

To compare SOSG signal evolutions, we first computed the slope of each sample intensity. Then, we compared these slopes with Kruskal–Wallis followed by Dunn post-hoc test. The significancy threshold was set to 0.01.

## 4. Conclusions

We demonstrated that is possible to have an energy transfer after light or X-ray excitation between Gd or Tb and Photofrin, in solution. We were also able to demonstrate that this energy transfer occurs when Photofrin is adsorbed onto the AGuIX NPs and leads to the production of ^1^O_2_.

In vitro and in vivo study will be performed in the near future. We believe that this strategy using both Photofrin and AGuIX NP excited by X-ray will enable improving the efficiency of PDT.

## Data Availability

Data obtained for this study can by obtain under reasonable request.
